# Supplementary material for: Shedding Light on the Interaction of Human Anti-Apoptotic Bcl-2 Protein with Ligands through Biophysical and in Silico Studies
Source: Int J Mol Sci. 2019 Feb 16;20(4):860. doi: 10.3390/ijms20040860 (PMC6413030; doi:10.3390/ijms20040860)

Supplementary Information:

Table S1. Potential energy and essential dynamics (ED) analysis of the MD simulations of the Bcl-2 complexes under study.

**Bcl2 and complex**	**Potential energy (kJ/mol)**	**ED 2D projection (nm^2)**
Unbounded physiological form	-739988	156.13
Venetoclax bounded physiological form	-652857	160.95
Unbounded chimeric form	-640409	119.88
Venetoclax bounded chimeric form	-570630	76.41

Table S2. List of Bcl-2 nsSNP with deleterious phenotype prediction. The nsSNP localization in Bcl-2 sequence is described and the correspondent predicted stabilities presented.

SNP ID	Substitution	Bcl-2 domain	Stability prediction
rs775404824	G8E	BH4	Decrease
rs540701354	D34Y	Disordered	Increase
rs1800477	A43T	Disordered	Decrease
rs551395951	H94P	BH3	Decrease
rs528042823	L97P		Decrease
rs751038951	F104S		Decrease
rs762635201	S105F		Inconclusive
rs763718170	S105P		Decrease
rs777784952	R129C	Disordered	Decrease
rs148811059	G203S	Disordered	Decrease
rs748122615	R207W	Disordered	Decrease
rs376149674	G233D	Disordered	Decrease

Table S3. Summary of results obtained from Protein sequence analysis (Residue positions are given in parenthesis, Blue color indicates residues influences the PTM process)

Parameters	Bcl2_WT	Bcl2_A43T	Bcl2_H94P	Bcl2_L97P	Bcl2_R129C	Bcl2_G8E	Bcl2_S105P	Bcl2_S105F	Bcl2_F104S	Bcl2_R207W	Bcl2_D34Y	Bcl2_G233D	Bcl2_G203S
**Physiochemical Properties**													
Molecular Wt (Da)	26265.89	26295.92	26225.87	26249.85	26212.84	26337.96	26275.93	26325.99	26205.79	26295.92	26313.98	26323.93	26295.92
Theoretical pI	6.75	6.75	6.7	6.75	6.49	6.5	6.75	6.75	6.75	6.49	7.13	6.49	6.75
Negatively charged residues	22	22	22	22	22	23	22	22	22	22	21	23	22
Positively charged residues	21	21	21	21	20	21	21	21	21	20	21	21	21
Instability index	51.63	50.83	52.44	50.51	51.63	53.15	51.32	50.83	53.56	51.14	51.28	51.99	53.83
Aliphatic index	78.03	77.62	78.03	76.4	78.03	78.03	78.03	78.03	78.03	78.03	78.03	78.03	78.03
GRAVY	-0.136	-0.146	-0.129	-0.159	-0.107	-0.149	-0.139	-0.121	-0.151	-0.121	-0.127	-0.149	-0.138
**Putative Post-translational modification sites**													
C-Mannosylation	Absent	Absent	Absent	Absent	Absent	Absent	Absent	Absent	Absent	Absent	Absent	Absent	Absent
N-Glycosylation	Absent	Absent	Absent	Absent	Absent	Absent	Absent	Absent	Absent	Absent	Absent	Absent	Absent
O-Glycosylation	Present (50,51,56,62,69,70,74,87)	Present (**43**,50,51,56,62,69,70,74,87)	Present (50,51,56,62,69,70,74,87)	Present (50,51,56,62,69,70,74,87)	Present (50,51,56,62,69,70,74,87)	Present (50,51,56,62,69,70,74,87)	Present (50,51,56,62,69,70,74,87)	Present (50,51,56,62,69,70,74,87)	Present (50,51,56,62,69,70,74,87)	Present (50,51,56,62,69,70,74,87)	Present (50,51,56,62,69,70,74,87)	Present (50,51,56,62,69,70,74,87)	Present (50,51,56,62,69,70,74,87)
C-Glycosylation	Absent	Absent	Absent	Absent	Absent	Absent	Absent	Absent	Absent	Absent	Absent	Absent	Absent
GPI modification	Absent	Absent	Absent	Absent	Absent	Absent	Absent	Absent	Absent	Absent	Absent	Absent	Absent
N-Myristoylation	Absent	Absent	Absent	Absent	Absent	Absent	Absent	Absent	Absent	Absent	Absent	Absent	Absent
S-Farnesylation	Present (229)	Present (229)	Present (229)	Present (229)	Present (229)	Present (229)	Present (229)	Present (229)	Present (229)	Present (229)	Present (229)	Present (229)	Present (229)
S-Geranylgeranylation	Present (229)	Present (229)	Present (229)	Present (229)	Present (229)	Present (229)	Present (229)	Present (229)	Present (229)	Present (229)	Present (229)	Present (229)	Present (229)
Prenylation	Absent	Absent	Absent	Absent	Absent	Absent	Absent	Absent	Absent	Absent	Absent	Absent	Absent
Acetylation	Absent	Absent	Absent	Absent	Absent	Absent	Absent	Absent	Absent	Absent	Absent	Absent	Absent
Phosphorylation (S/T/Y)	Present (24,70,87,167,56,74,96,132,28,180)	Present (24,70,87,167,**43**,56,74,96,132,28,180)	Present (24,70,87,167,56,74,96,132,28,180)	Present (24,70,87,167,56,74,96,132,28,180)	Present (24,70,87,167,56,74,96,132,28,180)	Present (7,9,24,70,87,167,56,74,96,132,28,180)	Present (24,70,87,167,56,74,96,132,28,180)	Present (24,70,87,167,56,74,96,132,28,180)	Present (24,70,87,**104**,105,167,56,74,96,132,28,180)	Present (24,70,87,167,56,74,96,132,28,180)	Present (24,**34**,70,87,167,56,74,96,132,28,180)	Present (24,70,87,167,56,74,96,132,28,180)	Present (24,70,87,167,56,74,96,132,28,180,**203**)
Tyrosine Sulfation	Absent	Absent	Absent	Absent	Absent	Absent	Absent	Absent	Absent	Absent	Absent	Absent	Absent

Sumoylation	Present (92-96)	Present (92-96)	Present (92-96)	Present (92-96)	Present (92-96)	Present (92-96)	Present (92-96)	Present (92-96)	Present (92-96)	Present (92-96)	Present (92-96)	Present (92-96)	Present (92-96)

Table S4. Time averaged structural properties obtained from MD simulation for Wild-Type (WT) of Bcl-2 and its variants

SNP ID	Substitution	RMSD (nm)	RMSF(nm)	Radius of Gyration(nm)	Intra-molecular hydrogen bonds	Trace of the diagonalized covariance matrix (nm^2)	Inter-molecular hydrogen bonds	Hydrophobic area (nm^2)	Hydrophilic area (nm^2)	Domain Distances (nm)					
										BH1-BH2	BH1-BH3	BH1-BH4	BH2-BH3	BH2-BH4	BH3-BH4
Bcl2_WT		0.362	0.186	1.830	145	184.579	467	73.794	63.576	1.345	1.199	1.843	1.48	2.105	1.435
rs551395951	Bcl2_H94P	0.244	0.141	1.802	144	110.994	455	70.865	61.439	1.382	1.27	1.818	1.48	2.153	1.466
rs528042823	Bcl2_L97P	0.288	0.203	1.816	139	210.218	481	74.519	65.744	1.33	1.301	1.838	1.511	2.061	1.41
rs1800477	Bcl2_A43T	0.358	0.17	1.819	146	156.194	470	74.064	64.228	1.353	1.344	1.793	1.465	2.081	1.452
rs777784952	Bcl2_R129C	0.295	0.174	1.802	142	167.427	475	73.788	63.537	1.341	1.343	1.82	1.477	2.083	1.415
rs775404824	Bcl2_G8E	0.408	0.194	1.830	143	206.089	473	73.195	63.982	1.338	1.322	1.871	1.468	2.098	1.451
rs763718170	Bcl2_S105P	0.345	0.165	1.808	139	155.571	477	72.831	63.421	1.344	1.351	1.836	1.46	2.082	1.423
rs762635201	Bcl2_S105F	0.406	0.215	1.825	139	243.526	463	72.830	64.586	1.383	1.1	1.804	1.405	2.087	1.756
rs751038951	Bcl2_F104S	0.377	0.196	1.824	143	199.994	466	74.655	62.612	1.384	1.357	1.859	1.325	2.073	1.591
rs748122615	Bcl2_R207W	0.280	0.175	1.818	145	166.199	466	75.956	62.912	1.342	1.365	1.855	1.465	2.093	1.458
rs540701354	Bcl2_D34Y	0.334	0.17	1.806	143	159.055	454	72.621	62.122	1.346	1.306	1.815	1.457	2.074	1.415
rs376149674	Bcl2_G233D	0.418	0.178	1.853	141	177.832	488	74.719	65.266	1.352	1.36	1.835	1.438	2.099	1.475
rs148811059	Bcl2_G203S	0.342	0.181	1.825	143	169.393	475	75.277	64.845	1.337	1.306	1.844	1.353	2.064	1.514

Table S5. List of putative and known inhibitors (Taxol) for FLD of Physiological Bcl-2 form. The putative inhibitors were identified from HTVS and docking analysis against Zinc Data base (Ranked according to CDRUG P-Value).

Zinc Accession No	Structure	Ligand interaction Diagram
**ZINC20149102**	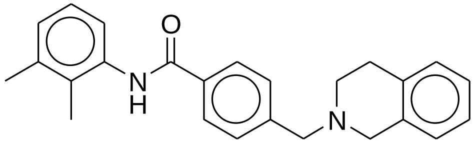	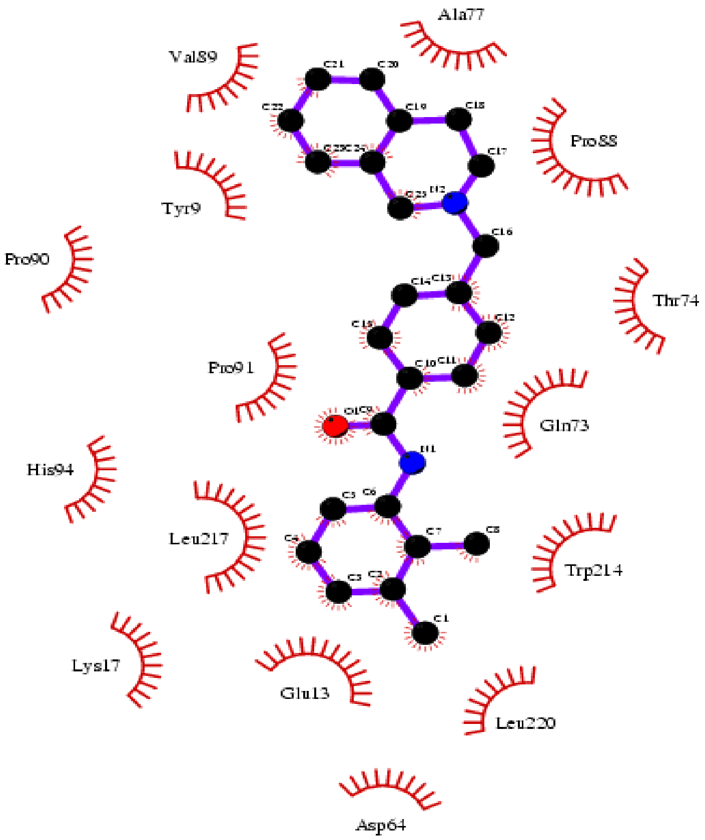

**ZINC22238492**	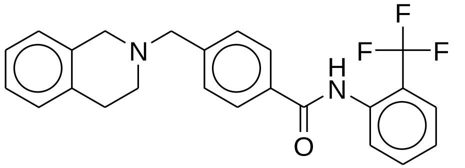	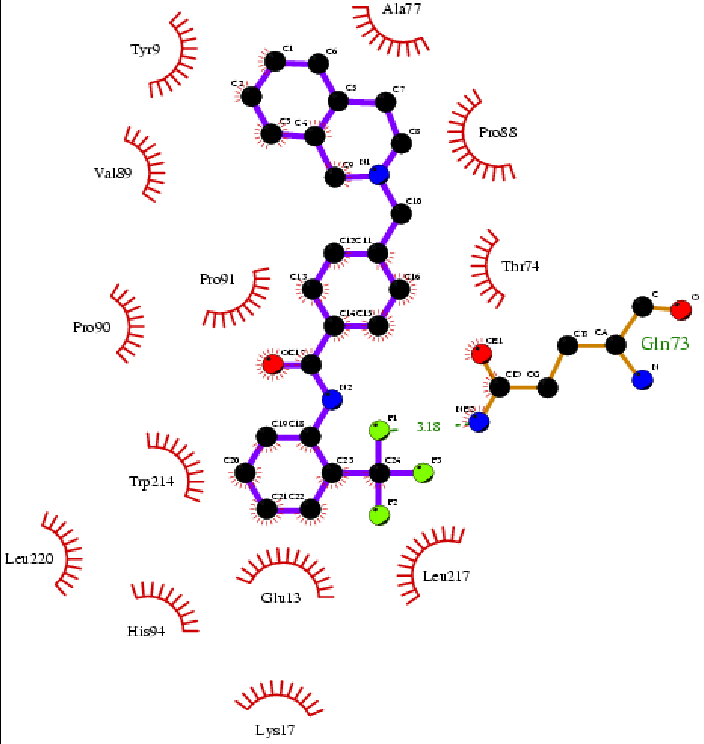

**ZINC09066116**	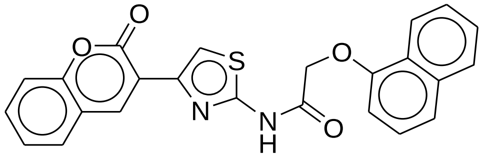	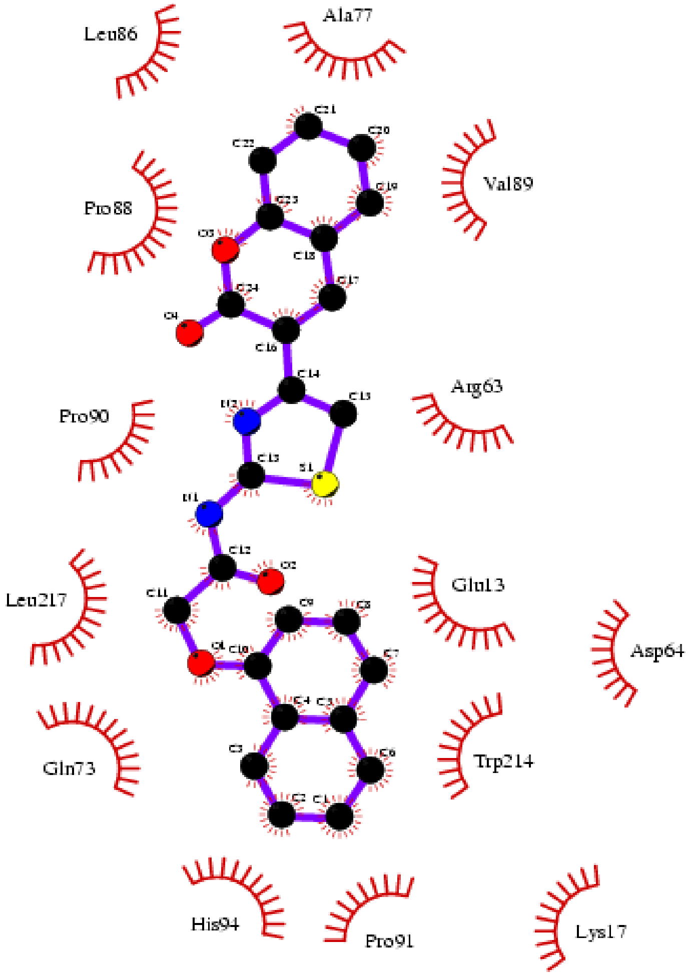

**ZINC04921974**	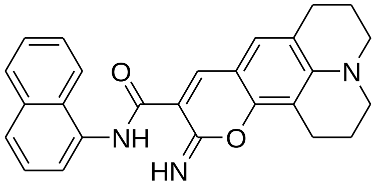	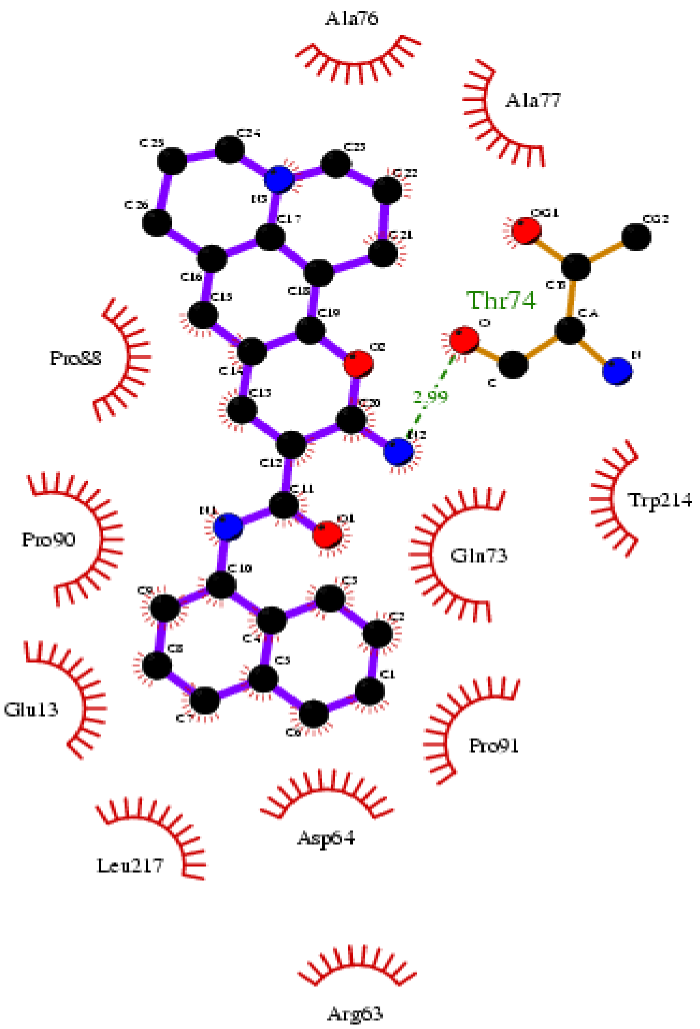

<table>
<tr><td>ZINC09475116</td><td>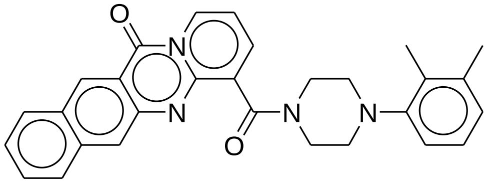</td><td>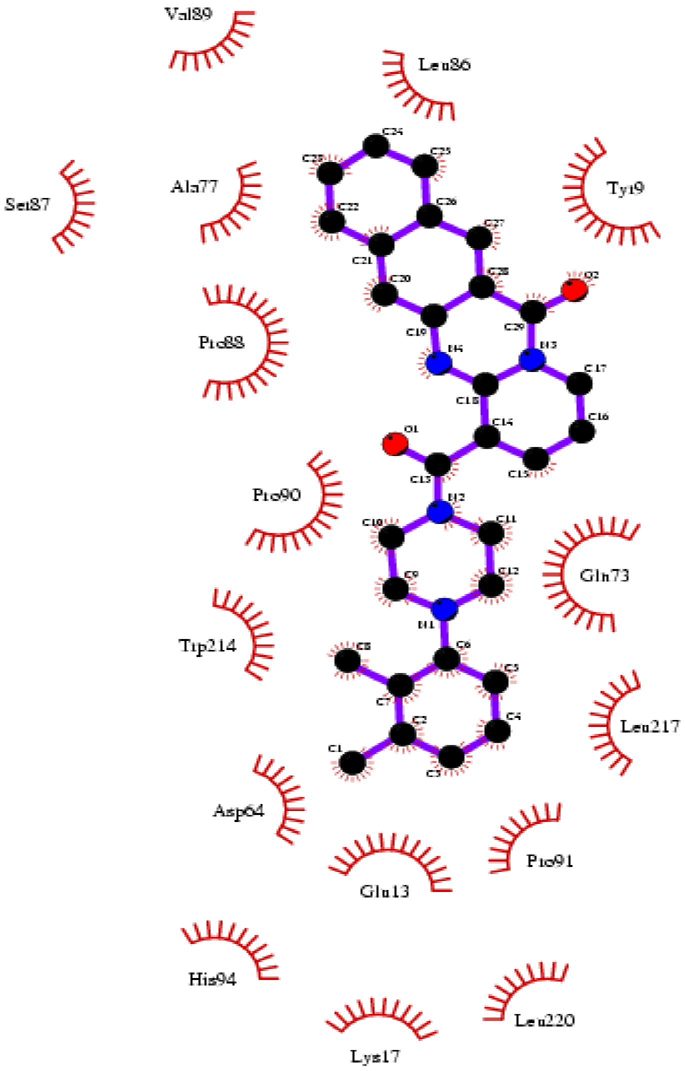
</td></tr>
</table>

**Taxol (ZINC96006020)**	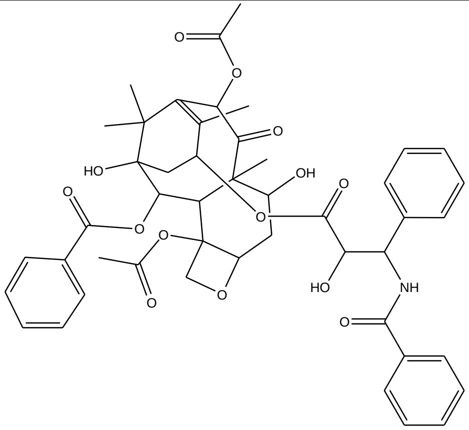	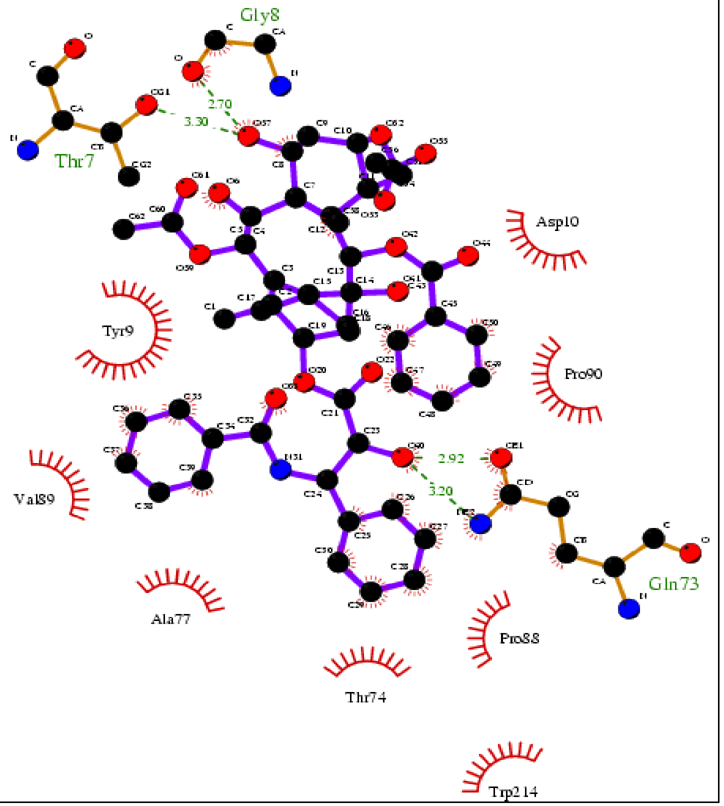

Table S6. Specific input parameters for MD simulations of each Bcl-2 nsSNP studied.

SNP ID	Substitution	SPC water molecules (#)	System net charge (e)	Ion replacement (# Na^+)
Wild-Type		15049	-1.00	1
rs775404824	G8E	15411	-2.00	2
rs540701354	D34Y	15419	0.00	0
rs1800477	A43T	15419	0.00	0
rs551395951	H94P	15364	-1.00	1
rs528042823	L97P	15364	-1.00	1
rs751038951	F104S	15423	-1.00	1
rs762635201	S105F	15416	-1.00	1
rs763718170	S105P	15366	-1.00	1
rs777784952	R129C	15418	-2.00	2
rs148811059	G203S	15414	-1.00	1
rs748122615	R207W	15415	-2.00	2
rs376149674	G233D	15415	-2.00	2

Figure S1. Molecular dynamics simulation results of Physiological Bcl2 form - Unbounded with Venetoclax bounded (a) Root Mean Square Deviation (Black: Apo & Red: complex), (b) Radius of Gyration (Black: Apo & Red: complex), (c) Potential energy (Black: Apo & Red: complex), (d) Principal component analysis (Black: Apo & Red: complex), (e) Solvent Accessible Surface Area for unbounded and (e) Solvent Accessible Surface Area for bounded forms

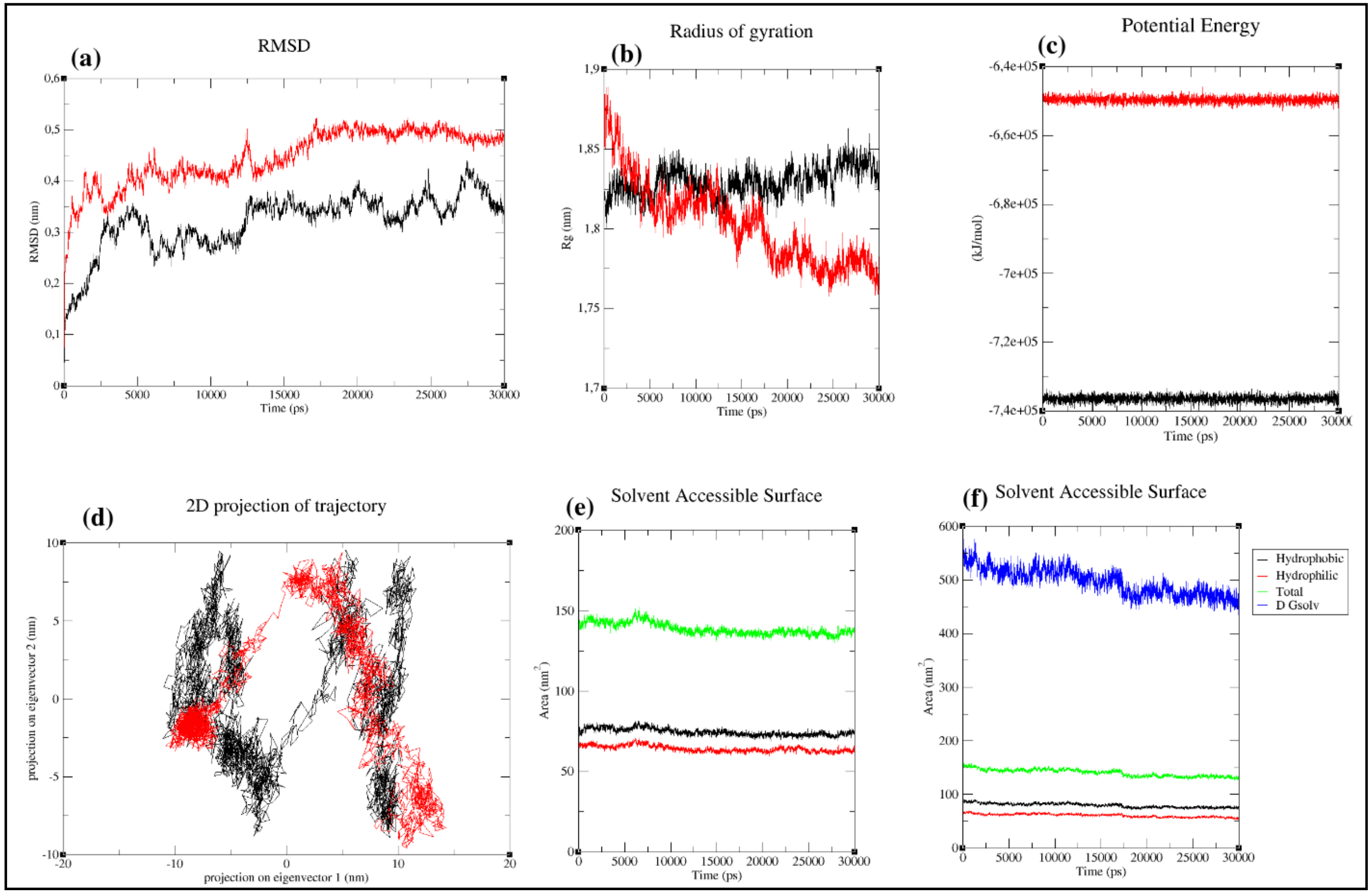

Figure S2. Molecular dynamics simulation results of Chimeric Bcl2 form - Unbounded with Venetoclax bounded (a) Root Mean Square Deviation (Black: Apo & Red: complex), (b) Radius of Gyration (Black: Apo & Red: complex), (c) Potential energy (Black: Apo & Red: complex), (d) Principal component analysis (Black: Apo & Red: complex), (e) Solvent Accessible Surface Area for unbounded and (e) Solvent Accessible Surface Area for bounded forms

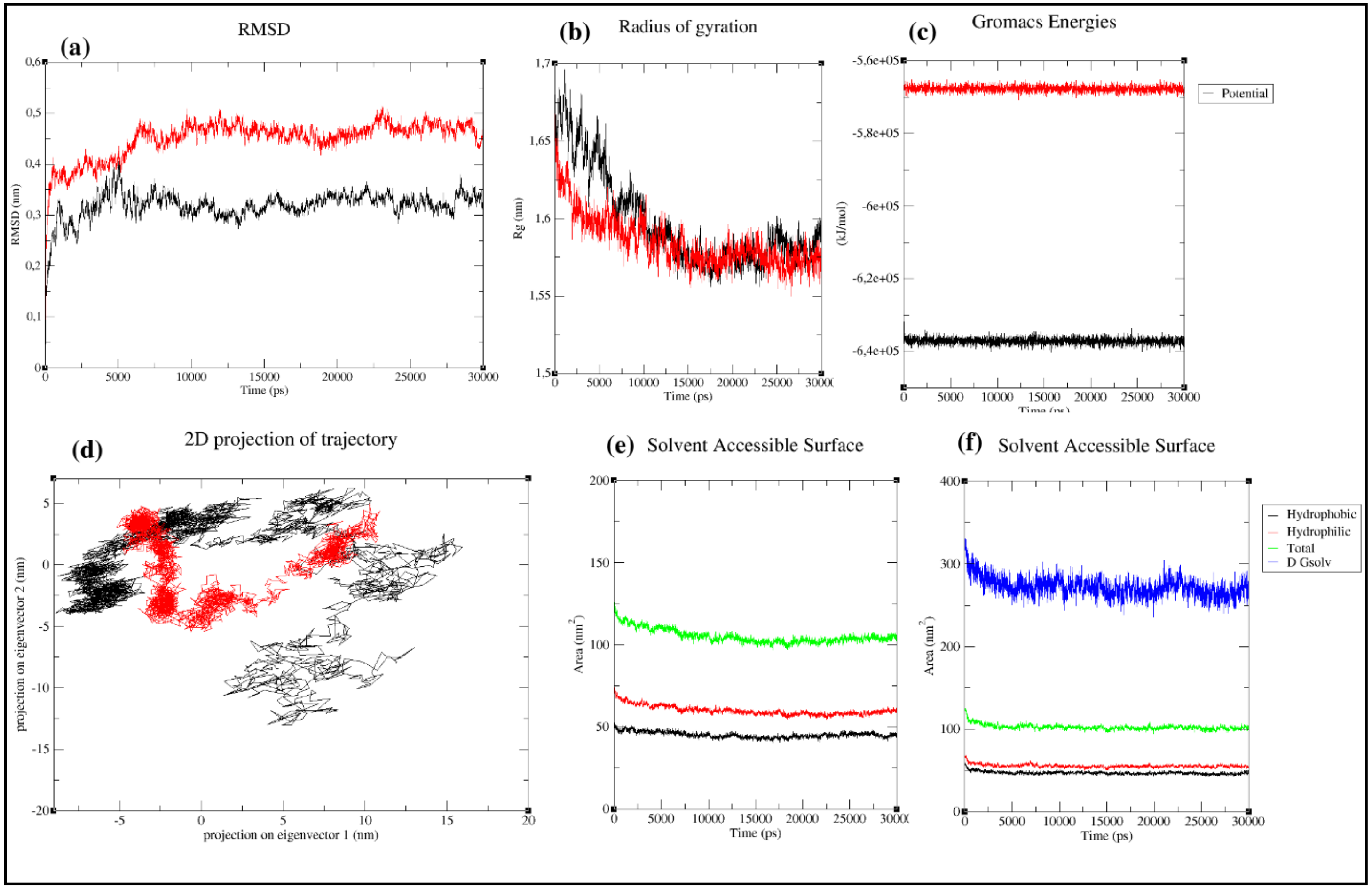

Figure S3. Molecular dynamics simulation results of Physiological Bcl-2 form:Venetoclax (Black) with Chimeric Bcl-2 form:Venetoclax (Red) (a) Root Mean Square Deviation, (b) Radius of Gyration, (c) Intermolecular hydrogen bonds, (d) Potential energy, (e) Principal component analysis, (f) Solvent Accessible Surface Area for Physiological Bcl-2 form:Venetoclax (g) Solvent Accessible Surface Area for Chimeric Bcl2 form:Venetoclax

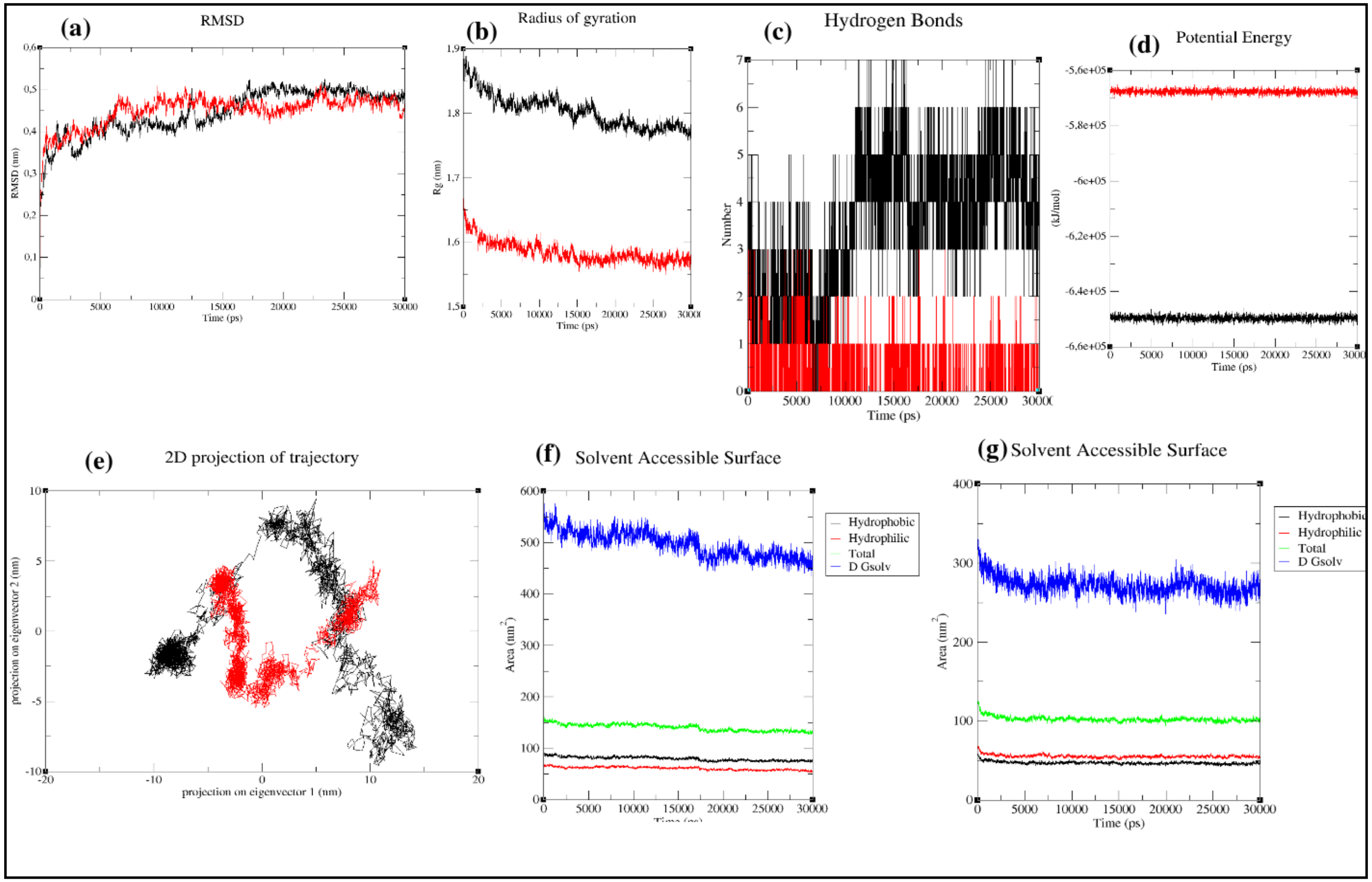

Figure S4. Surface representation of the residue occupancy probability of venetoclax towards (a) Chimeric and (b) Physiological Bcl-2 forms. The residues close to the ligand at less than 3 Å are represented in red, while the non-interacting residues are in gray. Representation was prepared by using PyMOL (PyMOL Molecular Graphics System, Version 1.8 Schrödinger, LLC).

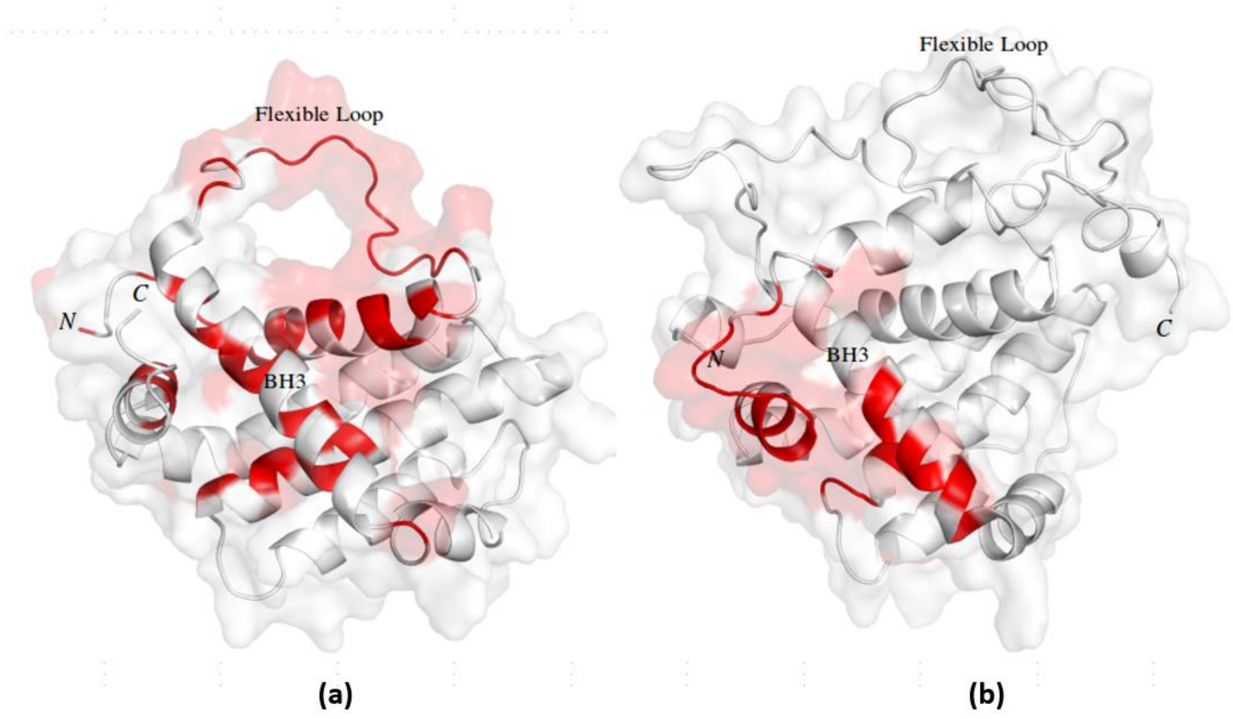

Figure S5. Structural alignment of Bcl-2 (PDB ID: 1GJH) with Bcl-XL (PDB ID: 2LPC). Chain 1 is Bcl-2 and chain 2 is Bcl-XL. Residues are color-coded by similarity match.

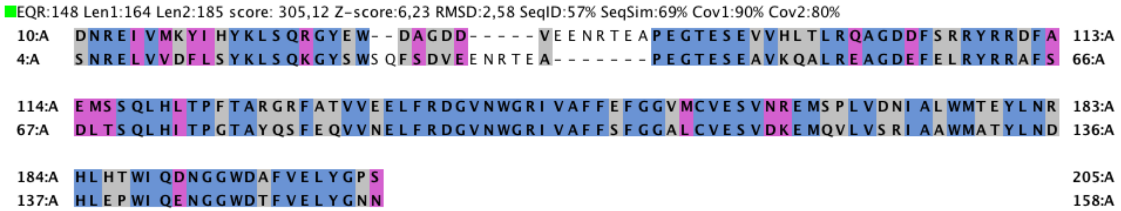

Figure S6. Structural alignment of Bcl-2 (PDB ID: 1GJH) with Bcl-W (PDB ID: 1MK3). Chain 1 is Bcl-2 and chain 2 is Bcl-W. Residues are color-coded by similarity match

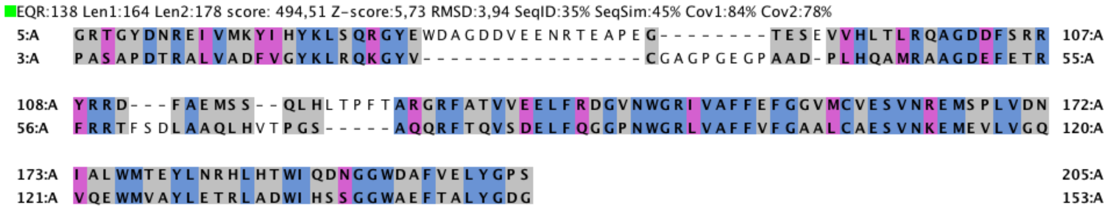

Supplement: Supplementary file 1 [file ijms-20-00860-s001.pdf]
